# Context-dependent effects of carbon dioxide on cross-modal integration during mosquito flight

**DOI:** 10.1038/s41598-025-13427-z

**Published:** 2025-08-20

**Authors:** Aya Kato-Namba, Kazumi Ohta, Takao Nakagawa, Hokto Kazama

**Affiliations:** 1 Human Health Care Research, Kao Corporation, 2-1-3 Bunka, Sumida-ku, Tokyo, 131- 8501 Japan; 2 RIKEN Center for Brain Science, 2-1 Hirosawa, Wako, 351-0198 Saitama Japan; 3 Graduate School of Arts and Sciences, The University of Tokyo, 3-8-1 Komaba, Meguro- ku, Tokyo, 153-8902 Japan

**Keywords:** Infectious diseases, Behavioural ecology

## Abstract

To navigate the environment and search for hosts, mosquitoes utilize multiple sensory cues including carbon dioxide (CO₂), visual, olfactory, and humidity cues. However, how mosquitoes shape their behavior by integrating these cues is poorly understood. Here we monitored the flight maneuvers of *Aedes albopictus* in a virtual reality environment where sensory cues were presented in open- or closed-loop. We found that CO₂ enhances optomotor responses to wide-field motion stimuli provided with various angular speeds and contrasts. Furthermore, CO₂ allowed more accurate tracking of a low contrast, visual object whose position was updated in closed-loop. We also found that olfactory preference is bidirectionally modulated by CO₂ depending on the value of odors: CO₂ enhances attraction to host odors whereas it enhances aversion to a repellent. This olfactory enhancement was further contingent on the humidity level. Together, our results highlight the context-dependent impact of CO₂ on cross-modal integration in mosquitoes.

**Supplementary Information:**

The online version contains supplementary material available at 10.1038/s41598-025-13427-z.

## Introduction

Mosquitoes are considered to be one of the most dangerous animals in the world as they transmit several infectious diseases that threaten human health including malaria and dengue fever. *Aedes* mosquitoes, specifically *Aedes aegypti* and *Aedes albopictus*, are major vectors of dengue, chikungunya, yellow fever, and Zika viruses, significantly increasing the global burden of infectious diseases^1^. Females of these mosquitoes feed on hosts to obtain proteins needed for egg development, during which they transmit pathogens. Therefore, avoiding mosquito bites through personal protection is pivotal to stay away from as well as to prevent the spread of mosquito-borne infectious diseases. Household measures for personal protection against mosquito bites include the use of insecticides and repellents. However, the emergence of insecticide-resistant mosquitoes on a global scale poses a significant challenge^2,3^and repellents must be applied in advance to repel mosquitoes. Consequently, there is a constant demand for new strategies to protect humans from mosquito bites, which necessitates gaining a better understanding of how mosquitoes navigate the environment and locate human hosts.

In a natural environment, mosquitoes heavily utilize CO₂ and visual cues to detect and approach humans. Mosquitoes are activated upon detection of CO₂^4,5^ in human breath^4^. CO₂ subsequently modulates mosquitoes’ steering responses to moving vertical bars^5^ and enhances attraction to visual features^6,7^ although dark contrast is inherently attractive^8^. It is also reported that CO₂ increases the sensitivity to visual contrast^9^. However, the effects of CO₂ on detailed flight patterns and wing kinematics in response to visual stimuli moving at various speeds and contrasts remain unclear. Moreover, how CO₂ affects the mosquitoes’ ability to actively track a visual object requires further investigation.

Odors such as those emitted from humans are another key stimulus that mosquitoes use to identify humans among other animals^10–12^. The interaction between CO₂ and odors is also crucial for optimizing host localization^13^. The effect of CO₂ on responses to aversive odors remains less understood.

The behaviors of mosquitoes have been commonly studied in Y-mazes, cages, wind tunnels, and greenhouses^11,13–16^. When combined with tracking of mosquito flight at high spatiotemporal resolution, these approaches offer significant advantages in examining the natural, unconstrained behavior of animals^6,7,13,16–18^. On the other hand, they are associated with difficulties in accurately assessing the sensory stimuli sampled by mosquitoes at each moment, especially for volatile compounds whose distribution and concentration fluctuate over time. Furthermore, it is not trivial to provide multiple sensory stimuli in various combinations in a controlled manner.

This challenge can be addressed by tethering mosquitoes and placing them in a flight simulator to observe their behavior in a controlled sensory environment, albeit with a caveat that the behaviors will be constrained to a certain extent. To date, studies using tethered mosquitoes have revealed aversive learning of body odor^19^interactions between vision and CO₂^7,10,11^, and modulation of visual behavior by sound^17^. However, these experiments typically involve mosquitoes responding passively to stimuli, which do not reflect behaviors in natural conditions where mosquitoes can actively control the interaction with the environment.

In this study, we developed a virtual reality environment where CO₂, odor, visual, and humidity^20–22^ cues were presented in various combinations with high spatiotemporal precision to examine cross-modal integration in mosquitoes. We investigated mosquitoes’ both passive and active interaction with sensory stimuli in open- and closed-loop systems. With this approach, we tested the hypothesis that multimodal cues are integrated in a context-dependent manner in flying mosquitoes.

## Results

### A multimodal virtual space for mosquitoes

**Fig. 1:**
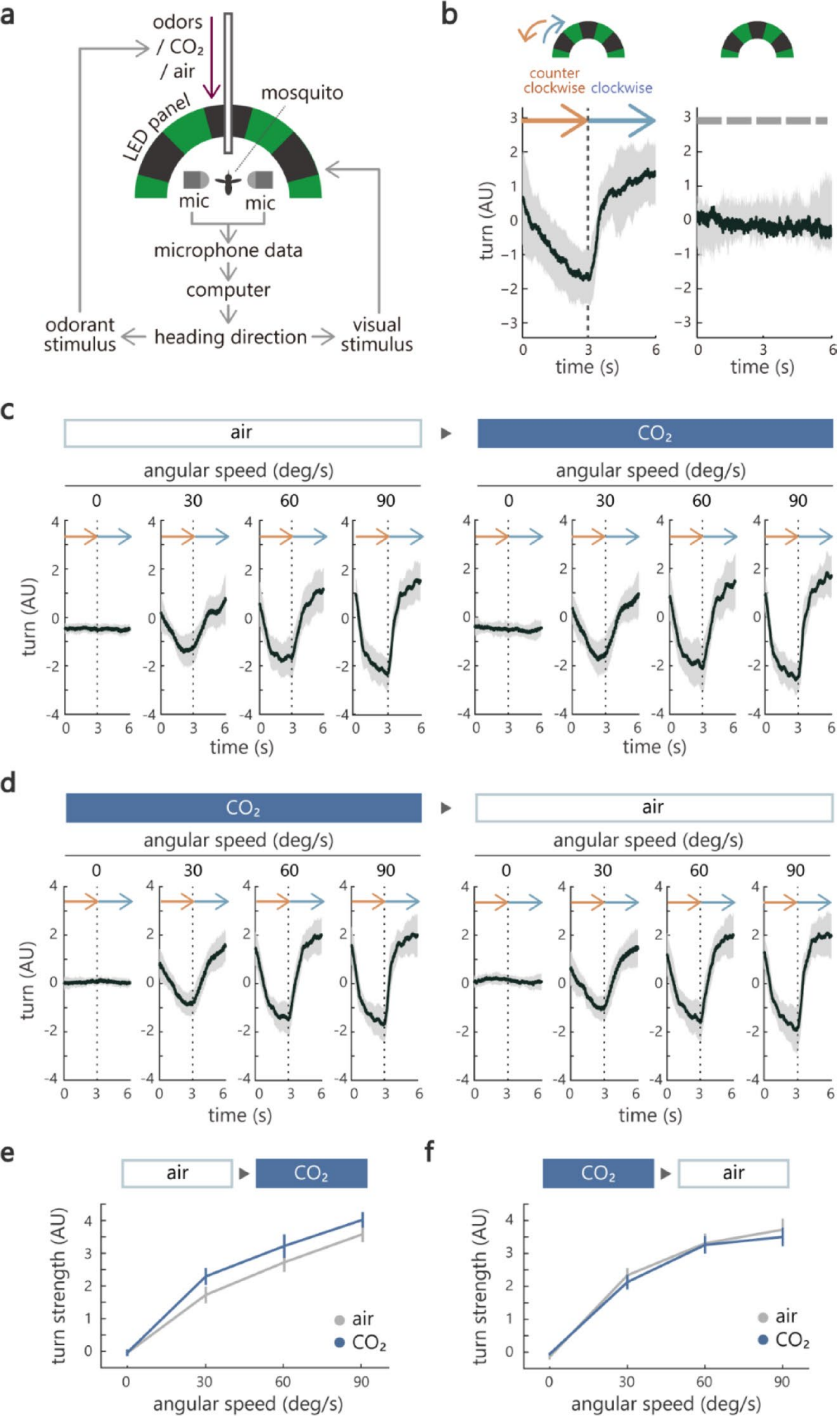
CO₂ enhances an optomotor response in mosquitoes. (**a**) Schematic of the virtual flight arena. Visual stimuli were presented with LED panels and odors/CO₂ were delivered from a tube facing the tethered mosquito. Stimuli were updated in open- or closed-loop. The flight behavior was analyzed using wingbeat amplitudes (WBA) and wingbeat frequency (WBF) recorded with a pair of microphones. (**b**) (left) Optomotor response to a vertical grating rotating counterclockwise and then clockwise for 3 s each at the speed of 90 deg/s. (right) Response to a stationary grating. The turning behavior (“turn”) was assessed by calculating the difference between left and right WBA (ΔWBA) over time (*n* = 10–12). (**c**) Optomotor response to a vertical grating rotating at various angular speeds. Individual mosquitoes experienced the same stimulus sequence twice, first in the absence and subsequently in the presence of CO₂ (*n* = 25). (**d**) The same as in (**c**), but for experiments where the order of CO₂ and air session was reversed (*n* = 21). (**e**) Turn strength (the difference between the peak turn value during counterclockwise and clockwise visual rotation) in (**c**). The turn strength is significantly higher under the presence of CO₂ (*p* = 0.018 and 6.0e-22 for air/ CO₂ and angular speed factors, repeated measures ANOVA, see Supplemental Table 1 for details and the results of post hoc multiple comparisons). (**f**) The same as in (**e**), but for the data shown in (**d**) (*p* = 0.46 and 1.4e-13 for air/CO₂ and angular speed factors, repeated measures ANOVA, see Supplemental Table 1 for details and the results of post hoc multiple comparisons). Black and gray lines show an average and standard error of mean across mosquitoes in (**b**), (**c**) and (**d**). Gray and blue plots correspond to data in air and CO₂ sessions, respectively in (**e**) and (**f**). Error bars represent standard error of mean in (**e**) and (**f**). “n” corresponds to the number of mosquitoes throughout the paper.

We examined the behavior of mosquitoes in a multimodal environment using the virtual flight arena previously developed to characterize the visual and olfactory behavior of fruit fly *Drosophila melanogaster*^23–25^ (Fig. 1a). The arena provides visual cues with an array of LEDs^26^ and olfactory cues from a tube placed in front of a tethered mosquito. We modified the arena to additionally provide CO₂ that releases attractive behaviors of mosquitoes^6,13,27,28^. The relative humidity of odors was set to high or low by passing the air through a gas washing bottle with or without water before delivering it to the olfactometer. Individual mosquitoes were placed between a pair of microphones that recorded the sound of the left and right wingbeats (Fig. S1). We operated the arena in either an open-loop condition where fixed sequences of sensory cues were presented, or in a closed-loop condition where the sensory environment was updated based on the mosquito’s behavior. More specifically, in the latter condition, the visual and olfactory environment was updated every 5 ms depending on the turning behavior assessed by the difference between the left and right wingbeat amplitude (ΔWBA: left – right wingbeat amplitude), a proxy for yaw torque^29^. Here, we termed ΔWBA as *turn* for simplicity.

In this study, we examined the flight maneuvers of mosquitoes under various contexts, beginning with visual stimuli, followed by addition of CO₂, the introduction of a closed-loop environment, addition of odors, and finally by modulating the humidity.

### CO₂ enhances an optomotor response

We first investigated a fundamental visual behavior called the optomotor response, the tendency for an animal to turn in the direction of a wide-field motion stimulus^30^. The optomotor response is crucial for stable course control during navigation as it allows animals to respond rapidly to sudden perturbations in their environment. We confirmed that mosquitoes expressed robust optomotor behavior in the virtual arena. When a wide-field grating was rotated counterclockwise and clockwise in open-loop, mosquitoes turned counterclockwise and clockwise respectively to track its movement (Fig. 1b). A stationary grating, however, did not induce consistent turns (Fig. 1b), indicating that the behavior was triggered by the movement of the visual stimulus.

We then examined whether and how CO₂ modulates this optomotor response. Previous studies on tethered mosquitoes provided mixed results reporting the presence or the absence of effects of CO₂ on responses to a rotating wide-field stimulus^5,9^. To examine the possibility that the effect is dependent on the angular velocity of visual stimuli, we characterized the impact of CO₂ while moving the grating at various velocities. We rotated the grating counterclockwise and clockwise at 0, 30, 60, or 90 deg/s. Each mosquito experienced the entire stimulus set twice, with or without the presence of 0.4% CO₂ (Fig. 1c, d and Fig. S1c, d), a concentration that evoked robust responses in the mosquito sensory neuron^31^. In the control (air) session, mosquitoes made stronger turns in the direction of visual motion as the angular velocity was increased (Fig. 1c). We found that the turns became even stronger under the presence of CO₂ (Fig. 1c). The difference between peak turn during counterclockwise and clockwise visual rotation, which we termed as *turn strength*, was significantly larger in the CO₂ session (Fig. 1e). Notably, when the order of sessions was reversed such that mosquitoes went through the CO₂ session first followed by the air session, we did not observe a difference in the turn strength (Fig. 1d and f), demonstrating that the effect of CO₂ persists for at least ~ 10 min (the length of a single session). It also indicates that the strengthened turns in the CO₂ session (Fig. 1c and e) are not merely due to plasticity such as motor learning. We noticed that the sum of left and right WBAs, which we termed *flight strength*, changed transiently when the direction of grating rotation was switched, although this was not statistically significant (Fig. S2).

Our results thus demonstrate that CO₂ enhances optomotor responses to a moving, wide-field stimulus.

### CO₂ enhances an optomotor response under low visual contrast

**Fig. 2:**
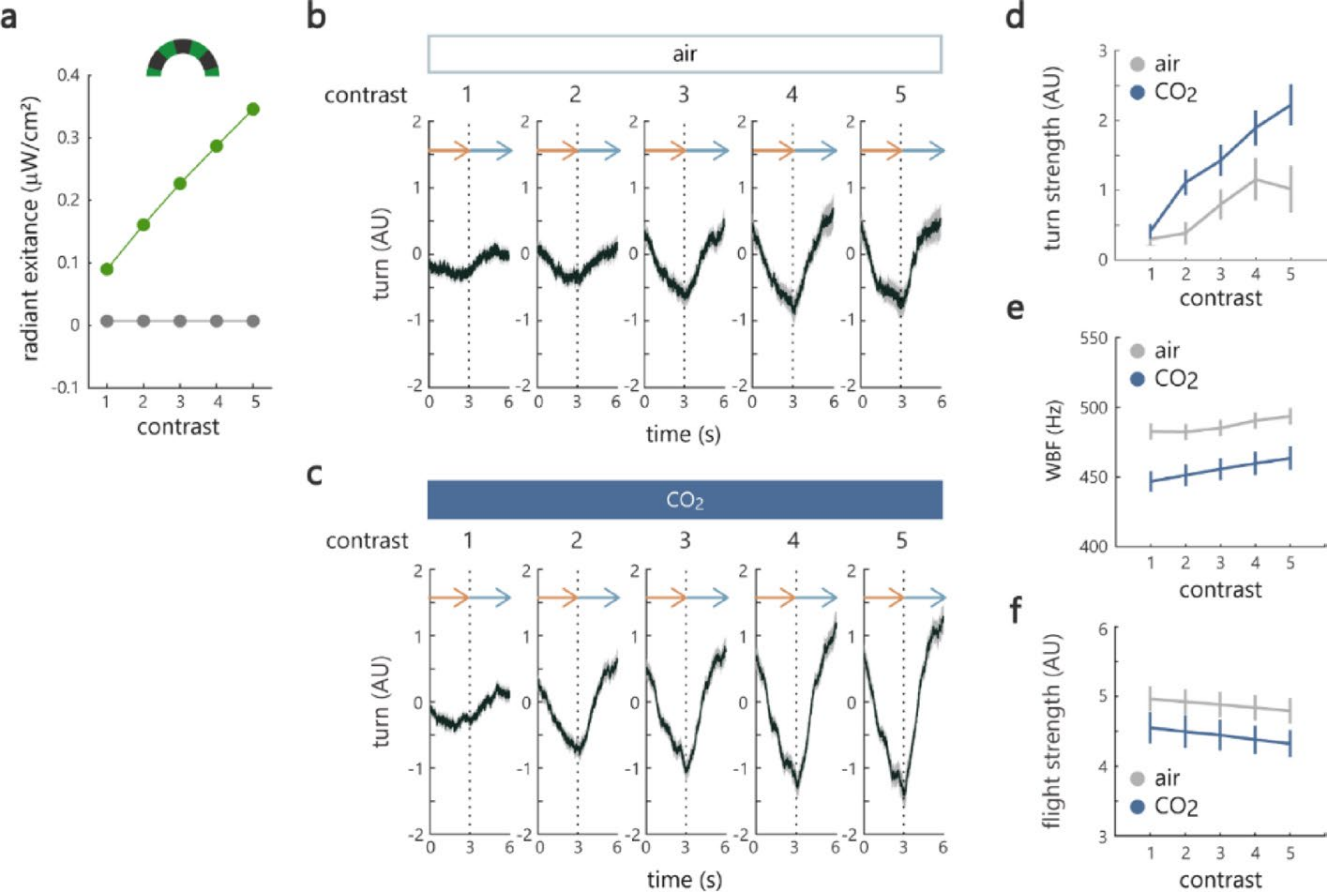
CO₂ enhances an optomotor response under low visual contrast. (**a**) Radiant exitance of a grating at 5 levels of visual contrast. Green and black plots represent the radiant exitance at bright and dark part of the grating. (**b**,**c**) Optomotor responses measured at 5 levels of visual contrast under the air (**b**) and CO₂ conditions (**c**) (*n* = 32 and 27 for air and CO₂). (**d**) Turn strength increased as a function of contrast and was higher under the presence of CO₂ (*p* = 5.6e-6 and 1.7e-8 for air/CO₂ and contrast factors, repeated measures ANOVA, the same data as shown in (b) and (c)). (**e**) WBF was lower under the presence of CO₂ (*p* = 3.6e-12 and 0.0015 for air/CO₂ and contrast factors, repeated measures ANOVA, the same data as shown in (**b**) and (**c**)). (**f**) Flight strength was lower under the presence of CO₂ (*p* = 0.00046 and 8.0e-5 for air/CO₂ and contrast factors, repeated measures ANOVA, the same data as shown in (**b**) and (**c**)). See Supplemental Table 1 for the details of statistics and the results of post hoc multiple comparisons for (**d**)–(**f**). Black and gray lines show an average and standard error of mean across mosquitoes in (**b**) and (**c**). Error bars represent standard error of mean in (**d**)-(**f**).

Because *Aedes* mosquitoes are active and bite humans even in dusk, we asked if CO₂ enhances behavioral responses to moving visual stimuli with low contrast. A recent study in *Aedes aegypti*, a close relative of *Aedes albopictus*, has shown that CO₂ decreases the contrast detection threshold^9^. We lowered the overall radiant exitance of LED by covering it with red filters and generated gratings at 5 different contrasts (Fig. 2a).

Based on our earlier observation that CO₂ exerts lasting effects, we let individual mosquitoes perform exclusively with or without the presence of CO₂, and made comparisons between the two groups of mosquitoes (air and CO₂ groups) in all of the following experiments. We found that the turn strength was stronger under the presence of CO₂ (Fig. 2b–d, *p* = 5.6e-06 for air/ CO₂ factor, repeated measures ANOVA, Supplemental Table 1) with a significant difference at a low contrast (post hoc multiple comparisons with Bonferroni correction, *p* = 0.02 for level 2, Supplemental Table 1). We also found that the wingbeat frequency (WBF) and the flight strength averaged at each contrast were lower in CO₂ condition (Fig. 2e, f, and Fig. S3). This suggests that mosquitoes increase turning while decreasing thrust to better follow the grating under the presence of CO₂. However, whether these changes correspond to an increase or decrease in the distance traveled sideways in a free flight setting is difficult to assess because of the limitation in inferring the relationship between wingbeat kinematics in the tethered preparation and the whole-body dynamics in free flight. Nevertheless, these results show that CO₂ modulates optomotor responses in a way that helps mosquitoes follow the whole-field visual motion even in low-contrast conditions.

### CO₂ enhances visual object tracking

These experiments revealed an effect of CO₂ on the optomotor response to whole-field visual motion, which is important for a mosquito’s flight control during navigation. Upon identification of a host, an ability to track the visual object also becomes crucial. However, this ability has not been rigorously examined as previous studies on tethered mosquitoes were mostly conducted in open-loop conditions where only passive visual responses could be quantified^5,8,9^.

**Fig. 3:**
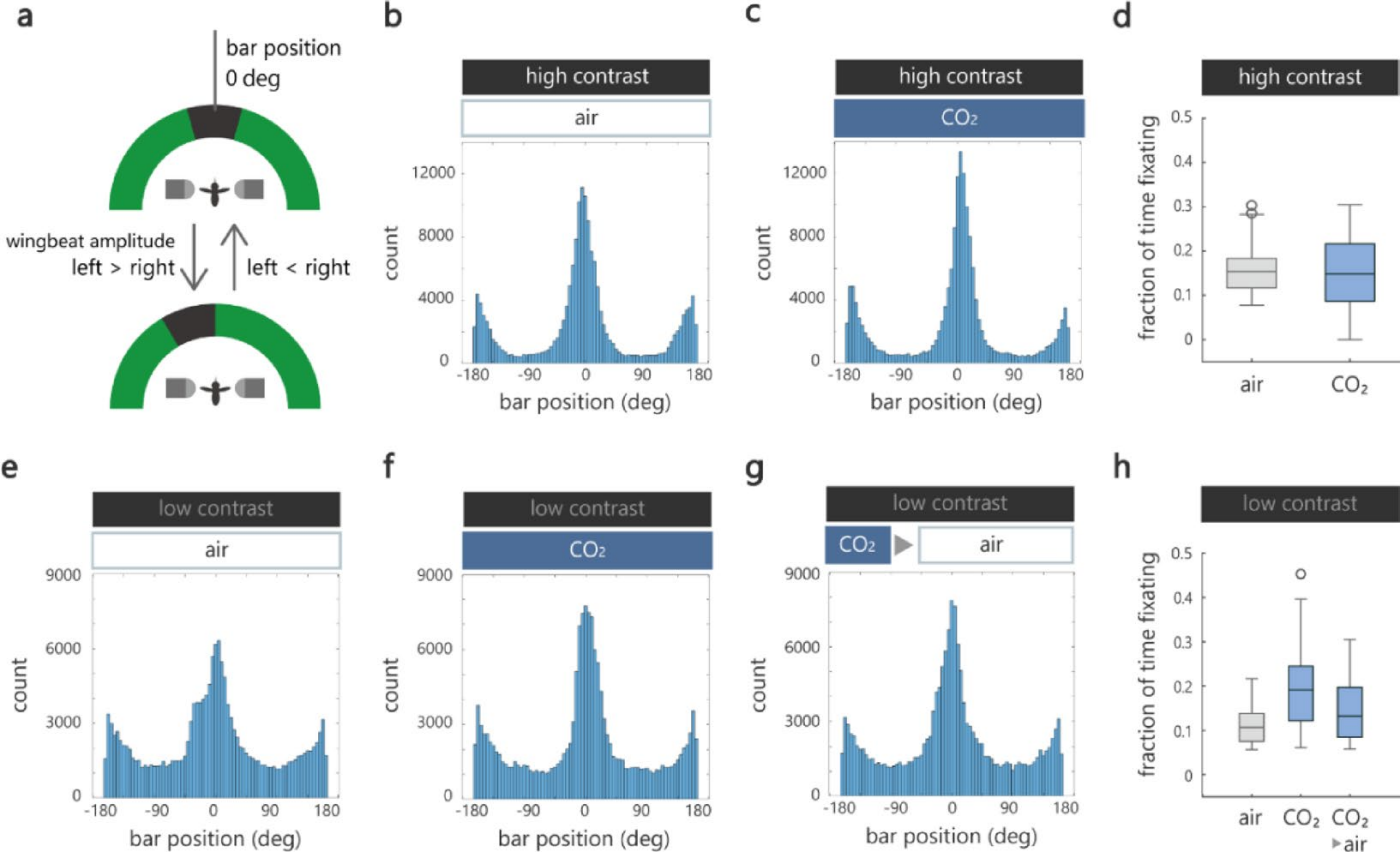
CO₂ enhances visual object tracking. (**a**) Schematic of the bar fixation assay. Two bars were presented in the arena, one in the front and the other in the back separated by 180 deg, whose positions were updated in closed-loop based on the mosquito’s turn. When the left WBA was larger than the right, the bar was shifted counterclockwise to reflect the mosquito’s effort to turn to the right, and vice versa. (**b**,**c**) Histogram of bar position, which reflects the accuracy of bar tracking, under the air (**b**) and CO₂ (**c**) condition. Each histogram was generated using the pooled data from 20 mosquitoes. (**d**) Fraction of time that mosquitoes fixated on the bar (by keeping the front bar within the frontal 10 degrees) calculated for individual mosquitoes. Because different mosquitoes fixated the bar at slightly different positions (meaning that the peak of the histogram was not always centered exactly at zero degree), we calculated the fraction over 10 degrees at the peak of the histogram. There was no difference between the air and CO₂ conditions (*p* = 0.87, t test, *n* = 20 and 19 for air and CO₂). (**e**,**f**) The same as in (**b**) and (**c**), but for experiments conducted under low visual contrast. (**g**) The same as in (**f**), but for the case in which CO₂ was applied for only 10 s just prior to the experiment. (**h**) Under lower visual contrast, fraction of time that mosquitoes fixated on the bar was larger under the presence of CO₂ (*p* = 0.033, one-way ANOVA, *n* = 15, 14, and 16 for air, CO₂, and 10 s CO₂ > air). Box plots in (**d**) and (**h**) represent median, quartile, nonoutlier range (see Methods for definition), and outliers (circles).

We therefore let the mosquitoes actively track a visual object whose position was updated in closed-loop according to their turning behavior (Fig. 3a). We hypothesized that this visual object tracking is enhanced with CO₂ as in the case of an optomotor response, because it should aid the mosquitoes’ ability to accurately approach a CO₂-emitting host. To test this, we set two bars separated by 180 deg, so that either bar was always present in the LED arena spanning ~ 210 deg. We assessed the accuracy of object tracking by plotting the histogram of bar position (zero deg corresponds to faithful tracking of the front bar) and calculating the fraction of time that mosquitoes kept the front bar within the frontal 10 degrees. Thus, a larger fraction indicates more accurate tracking. We found that CO₂ had little effect on bar tracking. Although the peak of the grand histogram incorporating the data from 20 mosquitoes was somewhat higher with CO₂ (Fig. 3b and c), the fraction of time that individual mosquitoes fixated on the bar was similar between the two conditions (Fig. 3d and Fig. S4).

We reasoned that the influence of CO₂ might have been minimal for tracking a high contrast bar (3.67 and 0.0042 µW/cm^2^ at bright and dark parts of the grating), which provided unambiguous information about the spatial position of a visual object. Indeed, nearly all the mosquitoes accurately tracked the bar most of the time even without CO₂ (Fig. S4). Therefore, we repeated the experiment with lower visual contrast (0.09 and 0.0071 µW/cm^2^ at bright and dark parts of the grating, corresponding to contrast level 1 in Fig. 2a). As expected, the accuracy of tracking became much lower (Fig. 3e and Fig. S5). Critically, CO₂ significantly increased the performance (Fig. 3f–h, and Fig. S5), indicating that CO₂ enhances object tracking when the salience of a visual cue is low.

It has recently been shown that a brief activation of CO₂-sensing neurons in female *Aedes aegypti* causes persistent behavioral changes lasting more than 10 min^32^. Therefore, we presented CO₂ only for 10 s just prior to the experiment rather than throughout the experiment. Although the effect was not statistically significant, again, the fraction of time that mosquitoes fixated on the bar tended to be higher compared to that without CO₂ (Fig. 3g, h and Fig. S6), indicating that a brief exposure to CO₂ persistently augments object tracking in mosquitoes.

### CO₂ differentially modulates olfactory behavior depending on the value of odors

Besides visual input, odors are also an important navigational cue for mosquitoes. Previous studies have reported attractive and aversive odors for mosquitoes as well as their interaction with CO₂ using Y-mazes, cages, wind tunnels or greenhouses^33–35^. However, how odors synergize with CO₂ to control mosquito behavior warrants further investigation in a more controlled setting.

**Fig. 4:**
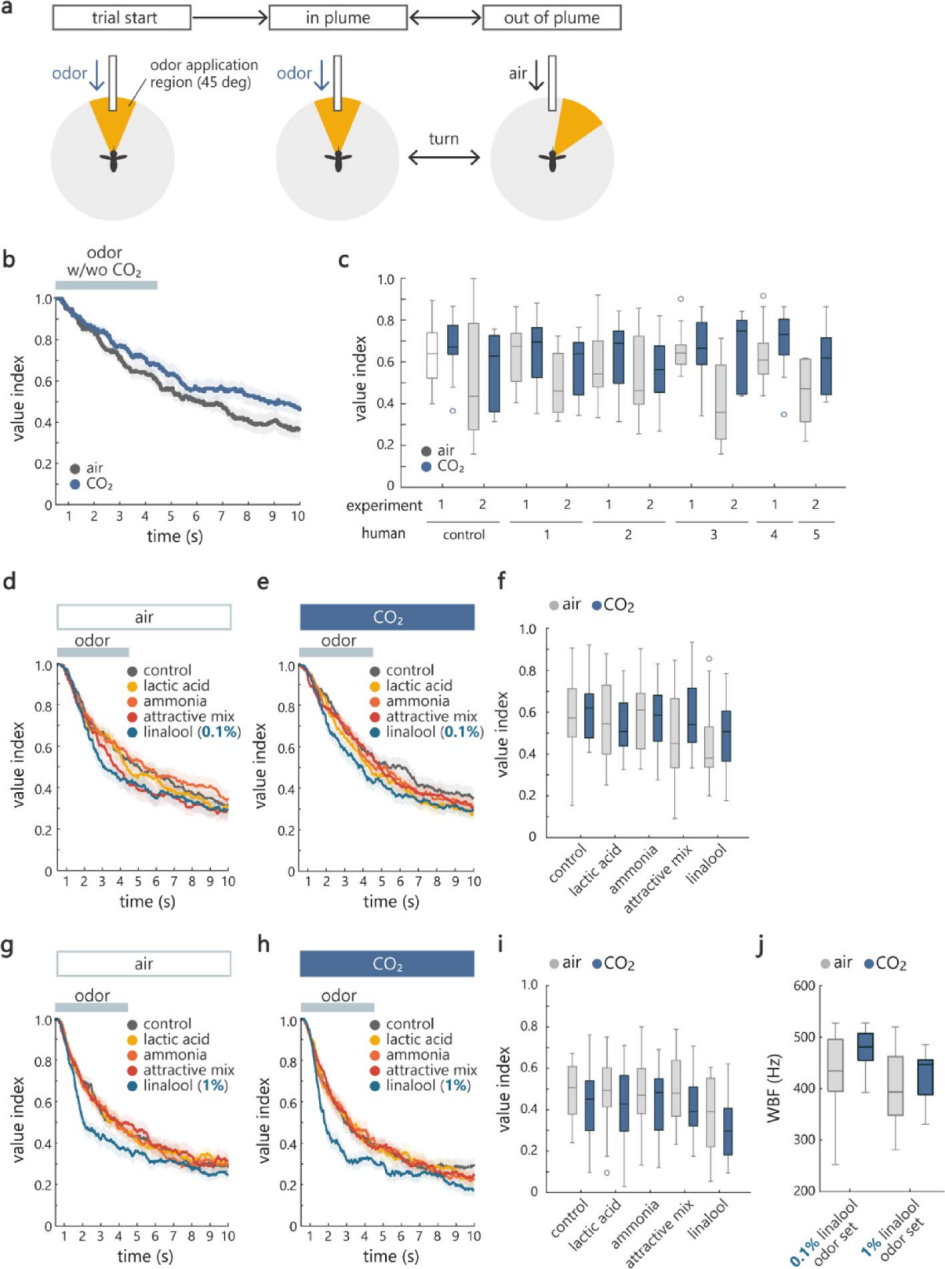
Context-dependent effect of CO₂ in multimodal environment. (**a**) Schematic of the olfactory behavioral assay. The mosquito started each trial in the center of the odor plume, after which it was free to adjust its heading direction to navigate in or out of the plume. (**b**) Instantaneous value index over time for an example human odor (experiment 2, human 2 in panel **c**) under the air and CO₂ conditions (*n* = 12 and 13 for air and CO₂ conditions). Value index is the proportion of time the mosquitoes spent inside the odor plume. At each time point, this proportion was calculated as a ratio of the number of trials in which the mosquitoes were within the odor plume to the total number of trials per experiment (15 trials). (**c**) Value index computed by averaging the instantaneous value index in the last 1 s of odor application period. Human odors were more attractive under the co-application of CO₂ (*p* = 0.01 and 0.06 for air/CO₂ and odor factors, two-way ANOVA). (**d**,**e**) The same as in (**b**), but for experiments with 5 synthetic odors under the air (**d**) and CO₂ (e) conditions (*n* = 20 for each condition). Instantaneous value indices during odor application period were not significantly different between odors for both conditions (*p* = 0.18 and 0.08 for air and CO₂ conditions, Scheirer–Ray–Hare test). (**f**) The same as in (**c**), but for data shown in (**d**) and (**e**). Value indices are different between odors but similar with or without co-application of CO₂ (*p* = 0.00093 and 0.31 for odor and air/CO₂ factors, repeated measures ANOVA). (**g**,**h**) The same as in (**d**) and (**e**), but for experiments where the concentration of linalool was higher (1%) (*n* = 20 for each condition). Instantaneous value indices during odor application period were significantly different between odors for both conditions (*p* = 0.003 and 8.8e-7 for air and CO₂ conditions, Scheirer–Ray–Hare test). (**i**) The same as in (**f**), but for data shown in (**g**) and (**h**). Value indices were different between odors and higher with co-application of CO₂ (*p* = 0.0001 and 0.0057 for odor and air/CO₂ factors, repeated measures ANOVA). (**j**) Average WBF was higher during the experiment where CO₂ was co-applied with odors (*p* = 0.03 and 0.0012 for air/CO₂ and linalool concentration factors, two-way ANOVA). See Supplemental Table 1 for the details of statistics and the results of post hoc multiple comparisons. Dark and translucent colors represent average and standard error of mean across mosquitoes in (**b**), (**d**), (**e**), (**g**) and (**h**). Gray and blue plots correspond to data in air and CO₂ condition, respectively, and box plots represent median, quartile, nonoutlier range (see Methods for definition), and outliers (circles) in (**c**), (**f**), (**i**) and (**j**).

We started by examining if mosquitoes show enhanced attraction to human odors in the presence of CO₂ in our virtual flight arena. Each mosquito sampled odors from four different individuals and air in random order, presented in the form of a plume extending 45 deg in azimuth (Fig. 4a). Odors were applied by passing air through a vial containing a piece of human-worn sock. We tested two sets of such human odors. Gratings were displayed on the LED to provide visual feedback. As odors were presented in closed-loop, mosquitoes were free to exit or re-enter the odor plume by making turns. Air was applied at the same flow rate outside of this odor zone to keep the mechanical information constant. We quantified the mosquito’s odor preference by calculating the proportion of time it spent inside the odor plume, which we termed *value index*. Therefore, a larger value index means that the mosquitoes are attracted by the odor whereas a smaller value index means that the mosquitoes have avoided the odor more actively by making turns. As expected, we found that mosquitoes were more attracted to odors under the presence of CO₂ (Fig. 4b) as assessed by the mean value index calculated during the last second of odor application period (Fig. 4c).

The properties of human odors can fluctuate depending on various factors including the physiological condition of individuals. To increase the reproducibility of experimental results as well as to characterize the behavioral responses to aversive odors, we subsequently used lactic acid, ammonia and a mixture of these two plus sulcatone as candidate attractive odors^11,33–35^and linalool as a candidate aversive odor^36^. It has been reported that lactic acid and ammonia are not attractive on their own, but lactic acid becomes attractive when it is presented together with ammonia or CO₂^33,34^. Sulcatone is linked to mosquito’s preference for humans^11^. We found that mosquitoes showed similar responses to all the odors tested. The value indices were similar across odors, including the air control (Fig. 4d). Salient odor preference was also not apparent under the presence of CO₂ (Fig. 4e), although linalool was more aversive than control when the data acquired under air and CO₂ were tested together (Fig. 4f). Notably, there were no differences in value indices between air and CO₂ conditions (Fig. 4f).

To examine whether linalool becomes aversive under each condition (air and CO₂) at higher concentration, we repeated the experiment with 1% instead of 0.1% linalool while keeping the other conditions the same. As expected, the value index of linalool decreased more rapidly over time and became distinct from that of other odors (Fig. 4g). A similar trend was observed under the presence of CO₂ (Fig. 4h). However, unexpectedly, the value indices of the other odors were also shifted downward in the CO₂ condition (Fig. 4i). Statistical analysis showed a significant difference not only across odors but also across conditions (Fig. 4i). These results obtained at two different concentrations of linalool demonstrate that mosquitoes change their overall behavior upon co-encountering a strongly aversive odor and CO₂. Moreover, together with the outcome of the experiments with human odors, CO₂ switches its effect depending on the value of odors present in the environment: it enhances attraction to host odors whereas it enhances aversion to a repellent.

### The effect of CO₂ on WBF depends on the experimental context

Mosquitoes did not change their WBF acutely in response to odors and CO₂ (Fig. S7). On the other hand, they did change their baseline WBF in the presence of CO₂. Intriguingly, the direction of change was variable depending on the experiment; CO₂ increased the baseline WBF in the olfactory experiment (Fig. 4j), did not exert a significant effect in the bar tracking experiment (Fig. S6b), and decreased it in the optomotor response experiment (Fig. 2e). These results suggest that the impact of CO₂ on WBF is dependent on the context, although we note the difficulty in directly comparing the behavioral parameter between open- and closed-loop conditions.

### Interaction among odors, humidity, and CO₂

**Fig. 5:**
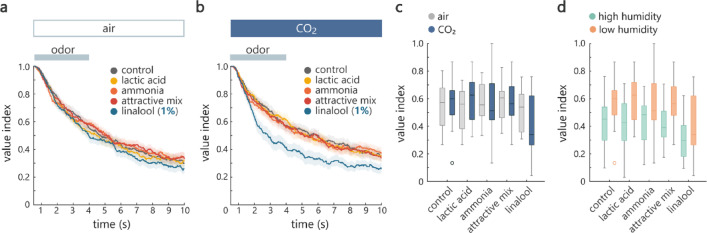
Interaction among odors, humidity, and CO₂. (**a**,**b**) The same as in Fig. 4g and h, but for experiments where the relative humidity was lower (13% as compared to 70% in Fig. 4). (**a**) was with air and (**b**) was with CO₂ co-application (*n* = 20 and 22 for air and CO₂ conditions). Instantaneous value indices during odor application period were significantly different between odors only under CO₂ condition (*p* = 0.67 and 0.028 for air and CO₂ conditions, Scheirer–Ray–Hare test). (**c**) Value indices were different between odors but not between conditions under low humidity (*p* = 0.00051 and 0.85 for odor and air/CO₂ factors, repeated measures ANOVA). (**d**) Value indices were lower under high relative humidity (*p* = 2.2e-5 and 4.4e-13 for odor and low/high humidity factors, repeated measures ANOVA, *n* = 20 and 22 for high and low humidity). The data was collected in CO₂ condition. See Supplemental Table 1 for the details of statistics and the results of post hoc multiple comparisons. Dark and translucent colors represent average and standard error of mean across mosquitoes in (**a**) and (**b**). Box plots in (**c**) and (**d**) represent median, quartile, nonoutlier range (see Methods for definition), and outliers (circles).

Finally, we added humidity to the context, which is a cue utilized by mosquitoes to survive, proliferate, locate hosts, suck blood and approach egg-laying sites^20–22^. How humidity interacts with odors and CO₂ to modulate mosquito behavior is unknown. To test this, we conducted experiments using the same odor set as in Fig. 4g-i, but after lowering the relative humidity of odors as well as air delivered during inter-stimulus-interval (see Methods). We found that mosquito’s aversion to high concentration linalool was absent under low humidity (Fig. 5a) unlike the case with high humidity (Fig. 4g). In contrast, aversion was observed when CO₂ was present (Fig. 5b), suggesting that CO₂ helps mosquitoes respond adaptively to aversive odors even under low humidity.

However, CO₂ did not restore all the behavioral aspects observed in high humidity. Although CO₂ decreased the overall value indices under high humidity (Fig. 4i), this was not observed under low humidity (Fig. 5c). Accordingly, when CO₂ was present, mosquitoes expressed stronger avoidance to odors under higher humidity (Fig. 5d). These results reveal interactions between odors, humidity, and CO₂ on mosquito flight maneuvers.

## Discussion

CO₂ is a sensory cue of particular relevance to mosquitoes. It triggers behaviors including take-off, flight, and landing near the CO₂ source^28^. It also increases the sensitivity to various stimuli such as human odors, visual cues, and temperature^5,6,9,13,27^. However, less is known about whether CO₂ changes the properties of mosquitoes’ sensory-cue-guided flight maneuvers. It also remains elusive how CO₂ functions in a richer multimodal environment, particularly where visual, olfactory, and humidity cues co-exist. Moreover, studies using tethered mosquitoes in a virtual space have focused on reactive responses to fixed stimuli provided in open-loop^5,8,9^precluding the investigation of actively controlled maneuvers in closed-loop. Here, we have addressed these issues in female *Aedes albopictus*.

The effect of CO₂ on an optomotor response has been variable across studies^5,9^hinting at a context-dependent action of CO₂. Although a study conducted at a particular visual contrast did not discern any effect^5^by exploring a range of angular speeds and contrasts, we found that CO₂ robustly enhances optomotor response in most of the stimulus space tested.

Whether CO₂ modulates the response to a single bar controlled in open-loop had also been controversial^5,8^. One study detected a change in turning maneuver under CO₂ that, according to simulation, aids object tracking^5^. Here we have directly proven this prediction by conducting a bar tracking experiment in closed-loop. Notably, the enhancement in the accuracy of object tracking was observed specifically when the visual contrast was low, demonstrating that CO₂ exerts an impact particularly when the visual stimulus is ambiguous. This likely helps mosquitoes target the host at dawn, dusk, or night. Nocturnal *Anopheles* mosquitoes integrate visual cues and airflow to escape looming threats, suggesting that cross-modal integration occurs between visual and other sensory modalities as well^37^. Similar investigations in nocturnal mosquitoes, such as *Culex* and *Anopheles*, should reveal how the visual systems of mosquitoes have evolved to utilize CO₂ to adaptively function under various contrasts.

In the olfactory domain, although CO₂ is frequently reported to synergize with attractive odors^13,27,33,34,38,39^less attention has been paid to its interaction with aversive odors^38^. In fact, it remains largely unknown whether CO₂, a stimulus of positive value to mosquitoes, counteracts or increases the salience of aversive odors. We found that while CO₂ enhanced attraction to human odors as previously reported, it enhanced aversion to a repellent, linalool, suggesting that it modulates behavior in different directions depending on the value of the co-applied odors. We further found that CO₂ did not simply increase the aversive responses to linalool, but also made the overall responses to odors in a stimulus set more aversive. Although linalool is associated with plants, this function might be useful for mosquitoes to escape from CO₂-emitting predators such as birds and bats. It will be interesting to test if mosquitoes perceive some odorants emitted from these predators as aversive, and whether escape maneuvers are augmented upon co-detection of CO₂. It awaits to be examined how CO₂ helps mosquitoes navigate toward the host while avoiding alerting entities in a complex multimodal environment.

The influence of CO₂ on olfactory behavior was further regulated by humidity. Although mosquitoes avoided linalool regardless of the presence of CO₂ under high humidity, they required CO₂ to express aversion under low humidity. Moreover, even with CO₂, mosquitoes did not change the overall aversiveness of odors when humidity was low. These results suggest that high humidity is preferable for mosquitoes in general; not just for survival and reproduction^21^ but also for navigation. Our results also reveal that the interaction between odors, CO₂, and humidity is layered and non-linear, suggesting a need for further exploration of this multi-modal space in the future.

What might be the neural mechanisms underlying cross-modal interaction between olfaction, vision and humidity sensation? In flies where more technologies are available to probe neural activity, several studies have addressed this issue centered around olfactory cues^40–44^. Olfactory information is integrated with airflow information in the fan-shaped body^42^visual information in the lobula plate^44^and gustatory information in the mushroom body^45^. In mosquitoes, multiple chemosensory receptors are expressed in the same neuron, showing non-canonical odor coding^46^. Therefore, the integration of CO₂, odor, and humidity information may take place in the peripheral nervous system. On the other hand, brain regions involved in chemosensory information processing, such as the antennal lobe, lateral horn, and subesophageal zone, as well as regions involved in visual information processing such as the fan-shaped body are larger in female mosquitoes compared to males^47^suggesting that CO₂ and visual information may be integrated in the central nervous system.

Reminiscent of the context-dependent (odor value-dependent) effect of CO₂ on olfactory maneuver, we found that CO₂ exerted differential effects on the overall flight speed in different experimental contexts; decreasing WBF in the experiment where mosquitoes needed to react to a moving grating (Fig. 2e) while increasing WBF in the experiment where mosquitoes explored the visual-olfactory space (Fig. 4j). Both of these changes may be adaptive as a decrease in WBF could allow mosquitoes to emphasize the turning maneuver to enhance an optomotor response, and an increase in WBF could allow mosquitoes to target odor objects more rapidly. At a shorter time scale, one study has reported a transient increase in WBF in response to a puff of CO₂^7^ whereas we did not observe this (Fig. S7). Because we applied CO₂ multiple times at a relatively short interval and because the influence of CO₂ exposure outlasts the stimuli^32^ (Fig. 1), WBF may have reached a plateau in our case. Another difference could have stemmed from the control that mosquitoes had over the duration of CO₂ and odor exposure in our closed-loop setting. Thus, it will be important to further scrutinize the mosquitoes’ flight in both open- and closed-loop environments.

In sum, we have shown that CO₂ controls mosquito’s flight by interacting with multiple sensory modalities. The effect of CO₂ on the behavior is diverse and occasionally even opposite depending on contexts with different visual contrast, odor value, and humidity. Despite this diversity, CO₂ generally appears to enhance visual and olfactory behavior, helping mosquitoes better explore the sensory environment. This suggests that manipulating CO₂ sensitivity alongside other sensory cues using a chemical or genetic approach may be an effective measure for regulating the behavior of mosquitoes.

## Methods

### Mosquito rearing and maintenance

All the experiments were performed on female *Aedes albopictus* 5 day after eclosion. Mosquitoes were reared at 28 °C, 70% relative humidity with a photoperiod of 12 h light and 12 h dark (lights on at 8 AM). Eggs were purchased from Sumika Technoservice Corporation and allowed to hatch in deoxygenated, deionized water. Larvae were fed TetraMin Baby (Spectrum Brands Holdings). Pupae were kept in deionized water contained in a small cup (Maruemu Corporation) placed within a plastic bottle (170 mL, AS-115, Thermo Fisher Scientific Inc.), and allowed to eclose. Female and male adult mosquitoes were kept in the same bottle for at least 2 days to let them mate. Mosquitoes were provided with unlimited access to 10 wt% sucrose solution (CAS: 57-50-1, nacalai tesque).

### Materials

Lactic acid (CAS: 50-21-5), ammonia (28% water solution, CAS: 1336-21-6), sulcatone (CAS: 110-93-0) and linalool (CAS: 78-70-6) were purchased from Tokyo Chemical Industry Co. Ltd. Lactic acid, ammonia and sulcatone were diluted to 10^− 6^ with water and applied either individually (lactic acid and ammonia) or as a mixture (lactic acid, ammonia, and sulcatone were mixed at equal ratio). Linalool was diluted to 10^− 2^ or 10^− 3^ with mineral oil. Human odors were collected by asking subjects to wear a pair of cotton socks for 8 h. A piece of sock that had covered the toes was placed in a glass vial and set in a custom-made olfactometer for odor application (see below). Human odors were used within two days and samples collected on different days were treated as different odors even if they were collected from the same subject. Odors were collected from 5 subjects (1 female and 4 males; odors 1 and 4, 2 and 5, 3 and 6 are from the same subjects collected on different days, and odors 4 and 8 are from different subjects).

### Mosquito Preparation for tethered flight

Mosquitoes were cold-anesthetized on ice, placed on a Peltier device held at 4 °C, and tethered to a stainless steel pin (Austerlitz minutiens Φ0.1 mm) on the thorax, using an ultraviolet-curing adhesive (NOA81, Norland). To prevent the mosquitoes from touching the microphones that collect the sound of wingbeats, their legs were removed at the base of femur before tethering them to a pin. Tethered mosquitoes were transferred to the virtual flight arena previously used to monitor the maneuver of *Drosophila melanogaster*^23–25^. The flight arena consisted of an odor delivery apparatus and a 24*56 array of green LEDs arranged in a half circle (Mettrix Technology Corp.). The display spanned ~ 210 deg horizontally and ~ 56 deg vertically below the mosquito. The entire arena was enclosed in an opaque container to prevent light entry, and the tethered mosquito was illuminated with infrared LEDs to allow visualization using a camera (Lu070M, Lumenera corporation).

### Olfactory stimulation

Odors were applied in the flight arena as described previously^23^. Briefly, air was odorized by passing it through 4 mL of odorant solution (using either mineral oil or water as a solvent) or a piece of human-worn sock (for human odor application) in a glass vial. The air stream was split into separate channels each lined up with a single odor vial and a solenoid valve (100E1-SR, Koganei Corporation) that regulated the open/closed state of the channel. The outputs of all channels were pooled. The stream of odors (250 mL/min) and the stream of pure CO₂ (8 mL/min, Leaf Corporation) were mixed into the main air stream (1.55 l/min), and directed into the arena using ⌀6 mm Teflon tubing (main tube). A small portion of this air stream was diverted from the main tube through a ⌀2 mm outlet tube and delivered frontally to the mosquito. This configuration reduced the impact of transient changes in air pressure caused by the switching of solenoid valves and was critical not to disturb flight at the onset of odor delivery. The tip of the delivery outlet was placed 10 mm away from the mosquito. The remainder of the air stream, which was not delivered to the mosquito, was directed out of the arena and cleared using a fume hood fan unit (3-4064-11, AS ONE). Because the quantity of air reaching the mosquito decreased with increasing the suction of the fan unit, suction power was calibrated to obtain an air flow of 0.3 m/s at the mosquito location, using an air flow sensor (QB-5, Tohnic). To avoid odor contamination, Teflon was used for all parts in contact with odorized air, and tubing was periodically washed with alcohol, rinsed with purified water, and dried with clean air.

The odor was presented in closed-loop by opening or closing the solenoid valve, which determines whether the air is passed through the odor vial or not, according to the turning behavior of the mosquito. When the valve for the odor was closed, the valve for the air was opened simultaneously to keep the total flow rate constant. Odors were provided only when the mosquito’s heading direction was within the restricted spatial region (45 deg centered at the mosquito’s heading direction at the time of odor onset). Odor application was terminated when the mosquito exited this spatial region but was re-initiated if the mosquito re-entered the region within the 4 s stimulus application period.

The humidity of air/odor applied to mosquitoes was measured by placing the hygrometer (Sensirion EK-H4) in front of the odor tube where the tethered mosquitoes were positioned during the experiments. The humidity of dry air delivered from the air tank (Suzuki Shokan) was 13% (relative humidity at 25 °C, low humidity condition). Humidity of air was increased to 70% (relative humidity at 25 °C, high humidity condition) by passing it through water contained in three gas washing bottles that were connected in series. Upon stimulation with odors where part of the air was passed through the vial containing odorant solutions, the humidity slightly increased or decreased depending on the solvent of odors (low humidity condition: no solvent (air) = 13%, water = 20%, mineral oil = 13%; high humidity condition: no solvent (air) = 70%, water = 73%, mineral oil = 69%). Because the amount of CO₂ added was small relative to the odor flow (8 mL/min vs. 1800 mL/min), the humidity did not change under the presence of CO₂. As the hygrometer was placed 10 mm away from the outlet of the odor tube (where tethered mosquitoes were placed during the experiments), the humidity measure changed by 2–3% depending on the relative humidity of the experimental room, which fluctuated throughout the year.

### Visual and olfactory behavioral experiments in a virtual flight arena

Visual and olfactory behavioral experiments were conducted using the same method applied to *Drosophila melanogaster*^23^. Mosquitoes’ flight was monitored using two microphones (AT9904 electret condenser microphones, audio-technica) positioned laterally ~ 1 mm from the tip of the extended wing on either side of the mosquito, whose outputs were amplified (AT-MA2 amplifier, audio-technica), digitized (NI 9215, National Instruments) and analyzed in real-time using a desktop computer (Optiplex 980, Dell) to extract the turning direction and speed, which were used to update the visual and olfactory stimuli in closed-loop. The visual display and solenoid valves were controlled using a data-acquisition board and modules (NI cDAQ9178, NI 9215, NI 9264, National Instruments) and custom code written in MATLAB (MathWorks), Java, and C. Microphone signals were acquired continuously in chunks of 5 ms, and signal amplitude in each chunk was computed as the difference between the maximum and the minimum values. These amplitudes were filtered by calculating the median over the 3 most recent values and (i) summed to obtain the flight strength s, and (ii) standardized (using mean and standard deviation values computed over the previous block of trials; for the first block, running estimates computed before the experiment were used) to obtain the standard left and right wingbeat amplitudes wL and wR. Turns during the flight were assessed by monitoring the difference in wingbeat amplitudes, a proxy for yaw torque^29^. An increment Δθ in angular position was registered if the difference in standard wingbeat amplitudes Δw = wR - wL, after multiplication by the flying strength, exceeded the value of its standard deviation σΔw (computed over the previous block of trials), above which the increment was proportional to Δw, i.e., ∆θ = γ sign(∆w) max(0,|∆w|s − σ∆w), with a coupling coefficient γ = 0.375 to yield units of degrees. This means that the angular position of the mosquito and thus the position of a visual stimulus was updated only when the mosquito exhibited a turn that exceeded a certain threshold. The same coupling coefficient was used in all the experiments. Angular position was computed by cumulatively summing these increments, and visual and olfactory stimuli were updated every 5 ms in accordance with the current position.

In the experiments assessing an optomotor response, a vertical grating with a spatial frequency of 60 deg^− 1^ was rotated counterclockwise for 3 s and then clockwise for 3 s in open-loop at the angular speed of 90 deg/s (Fig. 1b), 0, 30, 60 or 90 deg/s (Fig. 1c and f), or 60 deg/s (Fig. 2). This 6 s stimulus sequence was repeated 10 times (trials) consecutively under each condition, and the average turning behavior across trials was reported. For the assessment of angular speed tuning (Fig. 1c and f), individual mosquitoes went through both air and CO₂ sessions with one group starting from air and the other from CO₂ session. The inter-session-interval was ~ 1 min. Because the effect of experiencing CO₂ lasted for an extended period of time, individual mosquitoes performed exclusively with or without the presence of CO2 and comparisons were made between groups (air vs. CO₂) in all of the following experiments (Figs. 2, 3 and 4). For the assessment of visual contrast tuning (Fig. 2), green LED panels (peak wavelength is 568 nm) were covered with 2 sheets of color filter (Roscolux #39, Rosco) to reduce the overall radial exitance and a grating was presented at 5 different contrasts. This filter reduces the light intensity at this wavelength to about 1/100, but does not change the spectral composition as the transmission rate is flat at this wavelength range. Visual stimuli were presented in the order of ascending contrast. The radial exitance of LED (both on and off areas) at each visual contrast was measured with an optical power meter (Thorlabs, PM100D + S120VC).

In the bar tracking experiment, two 30 deg-wide bars separated by 180 deg were set in the arena at high or low contrast. The second bar was used so that either bar was always present in the LED arena spanning ~ 210 deg. Under this configuration, when one bar was positioned in the front (0 deg), the other bar in the back (180 deg) was not shown on the display. The initial position of the bars was random at the beginning of each experiment that lasted 870 s. The position of the bars was updated in closed-loop. CO₂ was presented either throughout (Fig. 3b, c and e, and 3g) or only for 10 s immediately prior to the experiment (Fig. 3f and g).

In the experiment involving odors (Fig. 4), the visual stimulus was a vertical grating with a spatial frequency of 60 deg^− 1^. The protocol consisted of 15 blocks, in which each of the 4 odors as well as a control odor (mineral oil) were applied in randomized order, up to a fixed duration (4 s) and in a restricted spatial region (45 deg centered at the mosquito’s heading direction at the time of odor onset). Odor application was terminated when the mosquito exited this spatial region but was re-initiated if the mosquito re-entered the region within the 4 s stimulus application period. Inter-trial interval was 10 s. When no odor was applied, air was presented in order to maintain the same flow rate. The random sequence of odor presentation was fixed across experiments and chosen not to present the same odor consecutively at the transition between successive blocks.

Experiments were initiated ~ 5 min after placing the mosquito in the flight arena, during which the position of the microphones were adjusted and the general behavior of the mosquito was observed. Mosquitoes flew in the presence of a wide-field grating controlled in closed-loop for ~ 1 min prior to the experiment. Occasionally, the air stream was turned on and off several times to trigger the flight. The experiment was terminated if the mosquito did not fly after ~ 5 min. Most of the mosquitoes flew continuously throughout the experiments and the percentage of time spent flying was 100% for optomotor experiments with varying visual contrasts (*n* = 59 mosquitoes) and 99.9 ± 0.2% for the olfactory experiments (*n* = 40 mosquitoes).

### Quantification and statistical analysis

All quantifications and statistical analyses were performed in MATLAB. Statistical details for each experiment, including the statistical test performed, data (dependent variables), analyzed factors, compared levels for post hoc multiple comparisons, ANOVA condition (within-subjects repeated measures, between subjects, or interaction), sample size, degrees of freedom, and p values are reported in Supplemental Table 1. The main results of statistical tests are also described in the corresponding figure legends. Sample sizes were set based on effect sizes and sample-by-sample variability observed in pilot experiments. For repeated measures ANOVA, the p value was adjusted with Greenhouse-Geisser method so that the assumption about compound symmetry can be relaxed. The p values for post hoc multiple comparisons were Bonferroni corrected. P values of less than 0.05 were considered as significant following the convention.

All the acquired data were included in the analyses except for a few cases where some data did not meet the pre-defined criteria. In the bar tracking experiments, mosquitoes that did not fixate on the bar at all were excluded. This involves the assessment of the flatness or the lack of a peak in the histogram of bar position (Figs. S4-S6). To this end, mosquitoes that did not seem to engage in tracking were tentatively chosen in a double-blind manner from part of the datasets collected under low visual contrast, and the mean and standard deviation of these histograms were calculated. The histogram was considered flat if its peak did not exceed the sum of mean and two standard deviations. This criterion was then applied to all the datasets to identify the mosquitoes that did not engage in bar tracking. This procedure did not exclude any mosquitoes in experiments conducted under high visual contrast (Figs. 3b-d and Fig. S4) but slightly reduced the number of mosquitoes (from 20 to 15 for control, 14 for CO₂ and 16 for CO₂ > air groups) in experiments conducted under low visual contrast (Figs. 3e-h and Figs. S5, S6). Although the statistical analysis of the level of bar tracking was conducted on this reduced dataset (Fig. 3d and h), for transparency, the histograms for all the mosquitoes are presented in Figs. S4-S6. In the odor response experiments (Figs. 4 and 5), 44/183,000 (0.024%) of the trials were excluded because the mosquitoes exhibited abnormal spinning behavior, continuously turning in the same direction for more than 720 deg. Also, 15/122 (12.3%) of the mosquitoes were excluded because they showed little turning behavior where the cumulative absolute turn within a trial (10 s) averaged across all trials and odors was less than 90 deg.

In the box plot, non-outlier range corresponds to 1.5 times the interquartile range away from the top or bottom of the box.

## Supplementary Information

Below is the link to the electronic supplementary material.

Supplementary Material 1

## Data Availability

The data and the code used in the current study are deposited in the CBS data sharing platform (10.60178/cbs.20250721-001).
