# Supplementary material for: Context-dependent effects of carbon dioxide on cross-modal integration during mosquito flight
Source: Sci Rep. 2025 Aug 20;15:28139. doi: 10.1038/s41598-025-13427-z (PMC12368251; doi:10.1038/s41598-025-13427-z)
Supplement: Supplementary file 1 — Supplementary Material 1 [file 41598_2025_13427_MOESM1_ESM.docx]

**Supplementary Information**
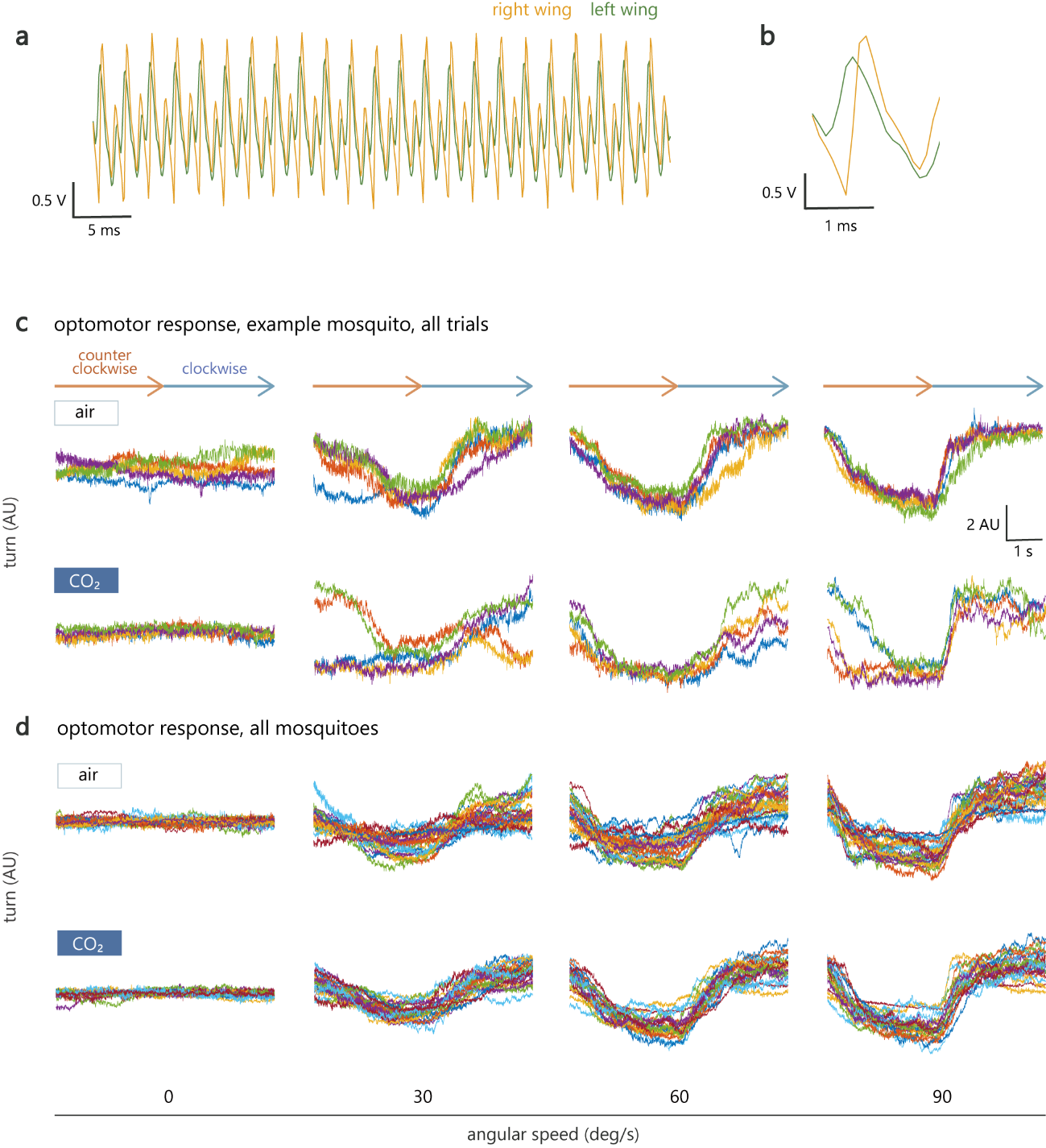


**Figure S1. Recording of wingbeats from a mosquito flying in the virtual flight arena, Related to Fig. 1**

(a) Raw microphone signals recording the sound of right (orange) and left (green) wingbeats of a mosquito flying in response to a grating rotating in a counterclockwise direction. The signal to noise ratio of recording was high enough to identify individual wingbeats. The right WBA is larger, reflecting a counterclockwise turn made by the mosquito.

(b) Enlarged view capturing a single wingbeat.

(c) Turn responses in the optomotor experiment in Fig. 1 for all the trials (5 trials), for example mosquitoes (one for air and one for CO_2_ condition), are superimposed to illustrate the typical trial-to-trial variability of the behavior. Different colors correspond to different trials.

(d) Averaged turn responses in the optomotor experiment in Fig. 1 for all the mosquitoes (n = 25 and 21 for air and CO_2_ conditions), are superimposed to illustrate the typical animal-to-animal variability of the behavior. Different colors correspond to different mosquitoes.


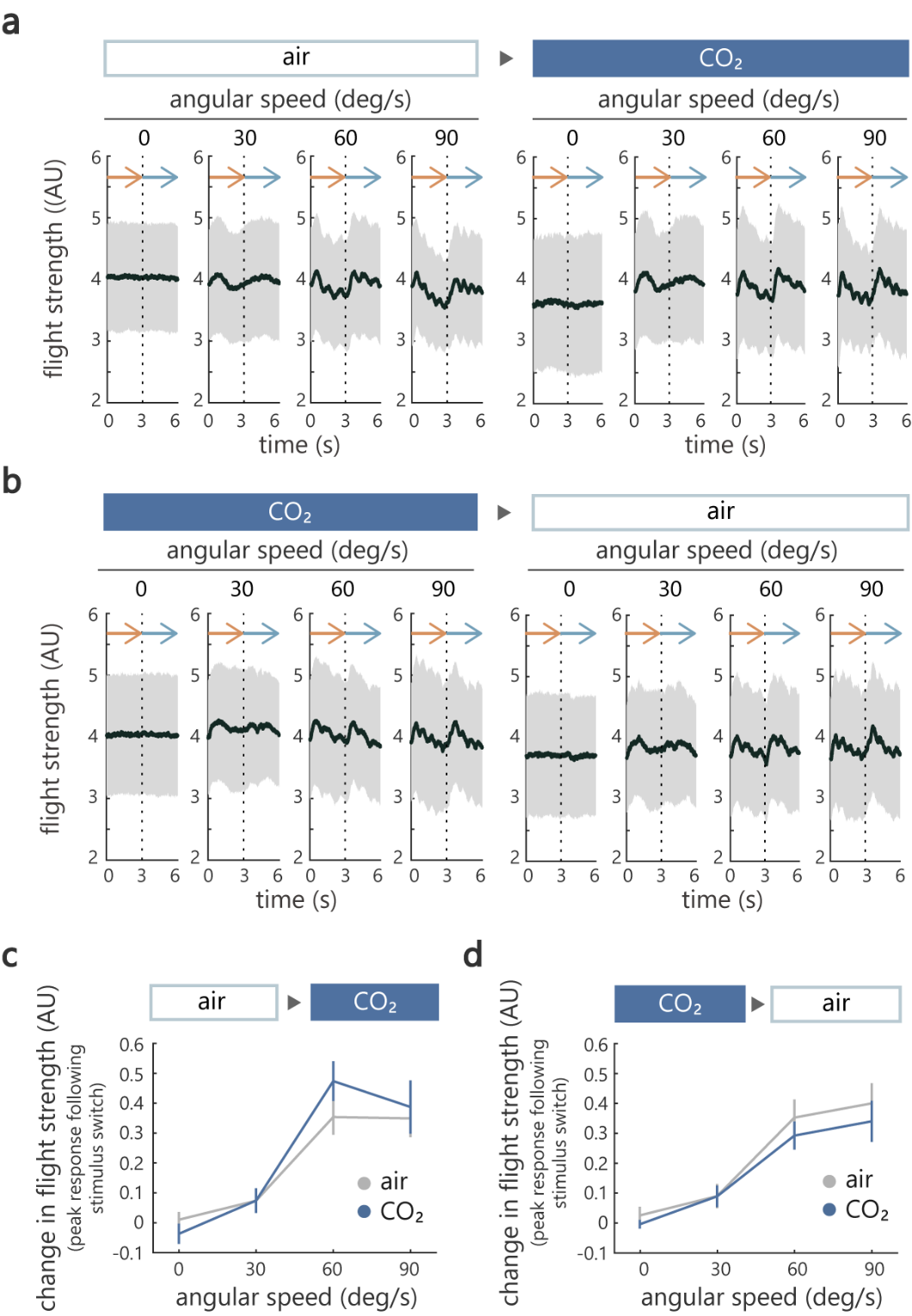


**Figure S2. Flight strength during optomotor responses, Related to Fig. 1**

(a,b) Same as in Figs. 1c and 1d, but for flight strength.

(c,d) Flight strength changed transiently when the direction of grating rotation was switched. The amplitude of this transient change was quantified for the transition from counterclockwise to clockwise rotation (average over 100 ms spanning the peak minus the average over 100 ms just after the transition in rotation). This value was not significantly modulated by CO_2_. See Supplemental Table 1 for the details of statistics.


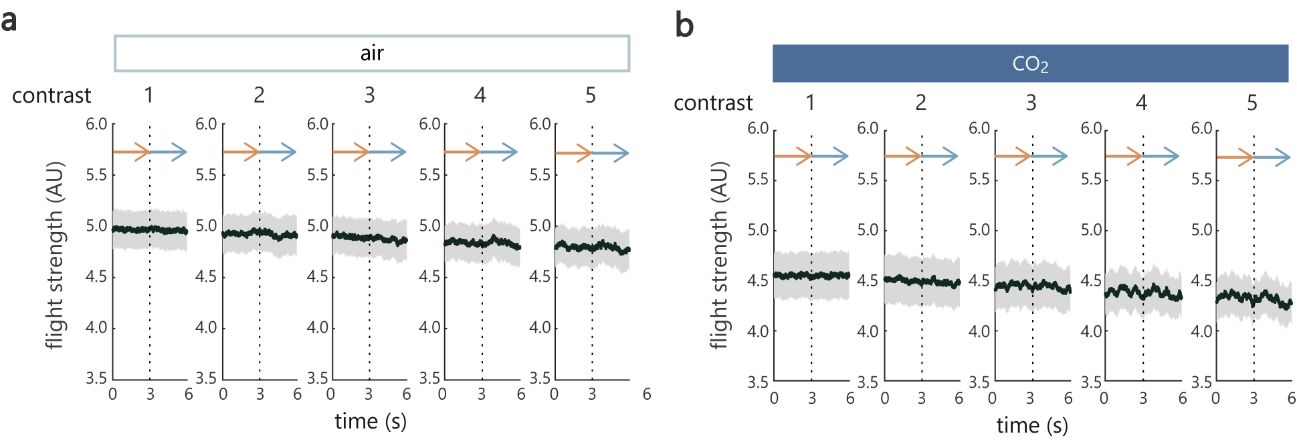


**Figure S3. Flight strength during optomotor responses, Related to Fig. 2**

(a,b) Same as in Figs. 2b and 2c, but for flight strength. Flight strength was lower in the presence of CO_2_. See Fig. 2f and Supplemental Table 1 for the details of statistics.

**
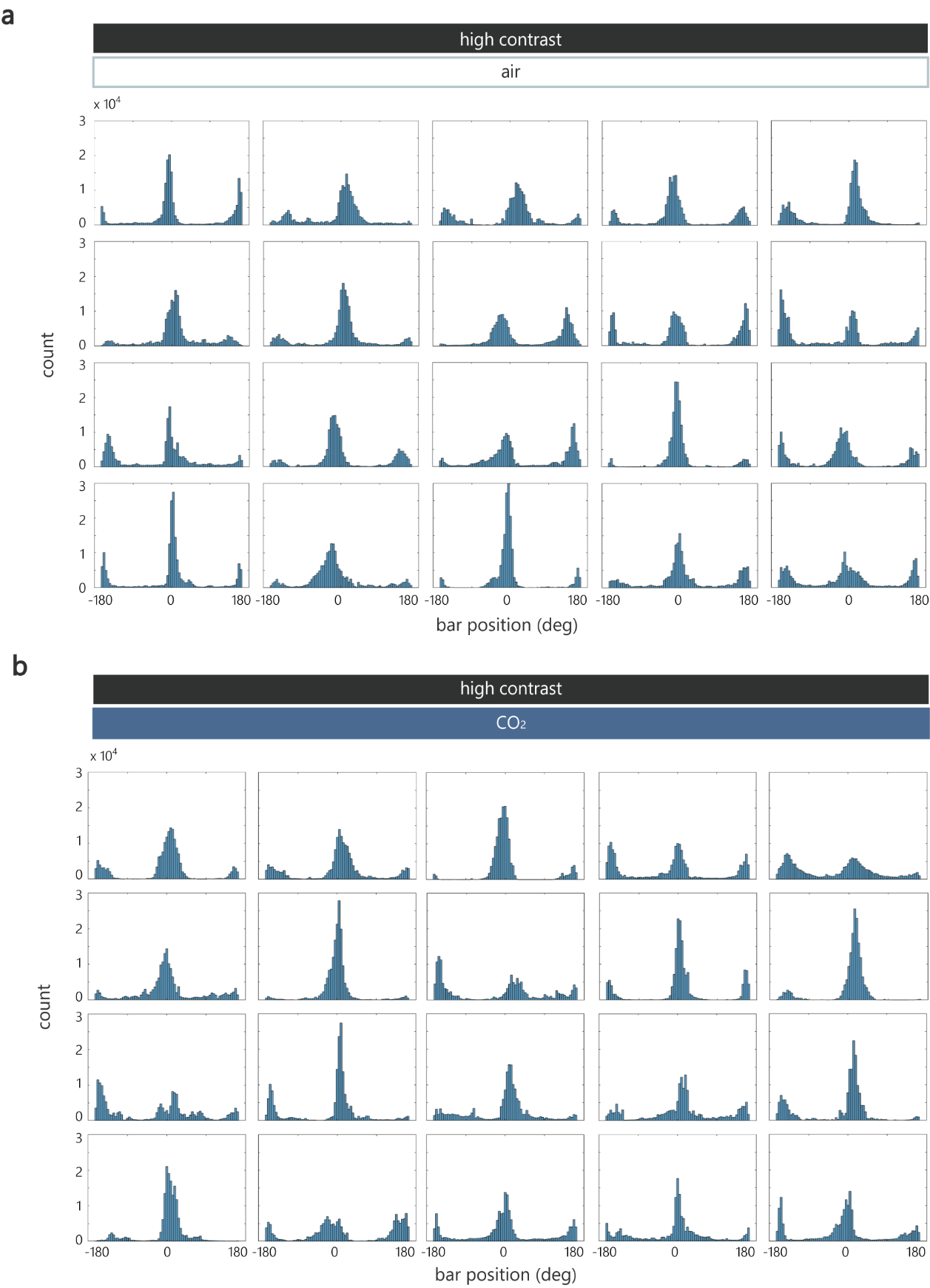
Figure S4. Histogram of bar position under high visual contrast, Related to Fig. 3**

Histogram of bar position for 20 mosquitoes under the air (a) and CO_2_ (b) condition. The data are from the same mosquitoes as in Fig. 3b and 3c.

**
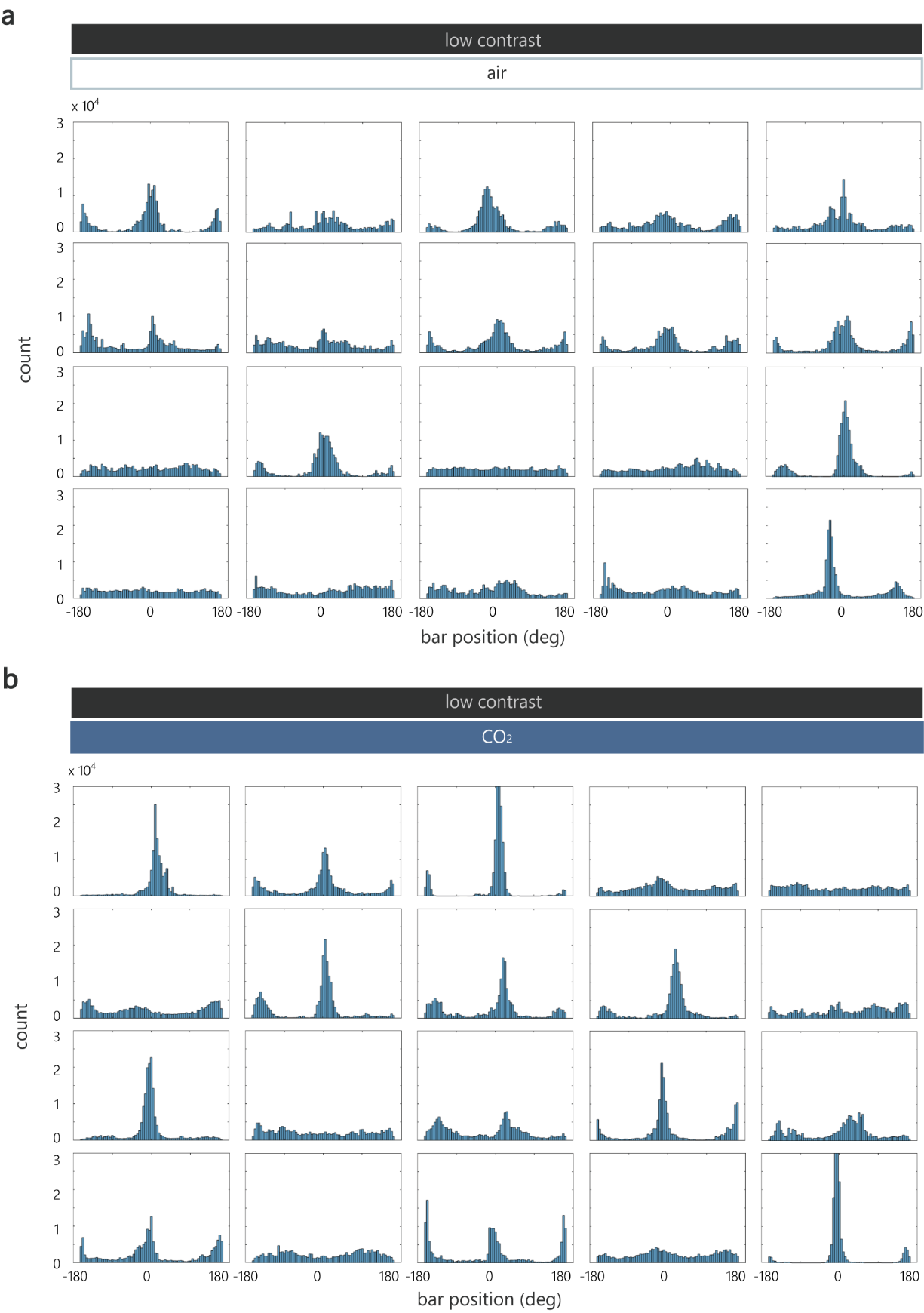
Figure S5. Histogram of bar position under low visual contrast, Related to Fig. 3**

Histogram of bar position for 20 mosquitoes under the air (a) and CO_2_ (b) condition, where the experiments were conducted under low visual contrast. The data are from the same mosquitoes as in Fig. 3e, f.


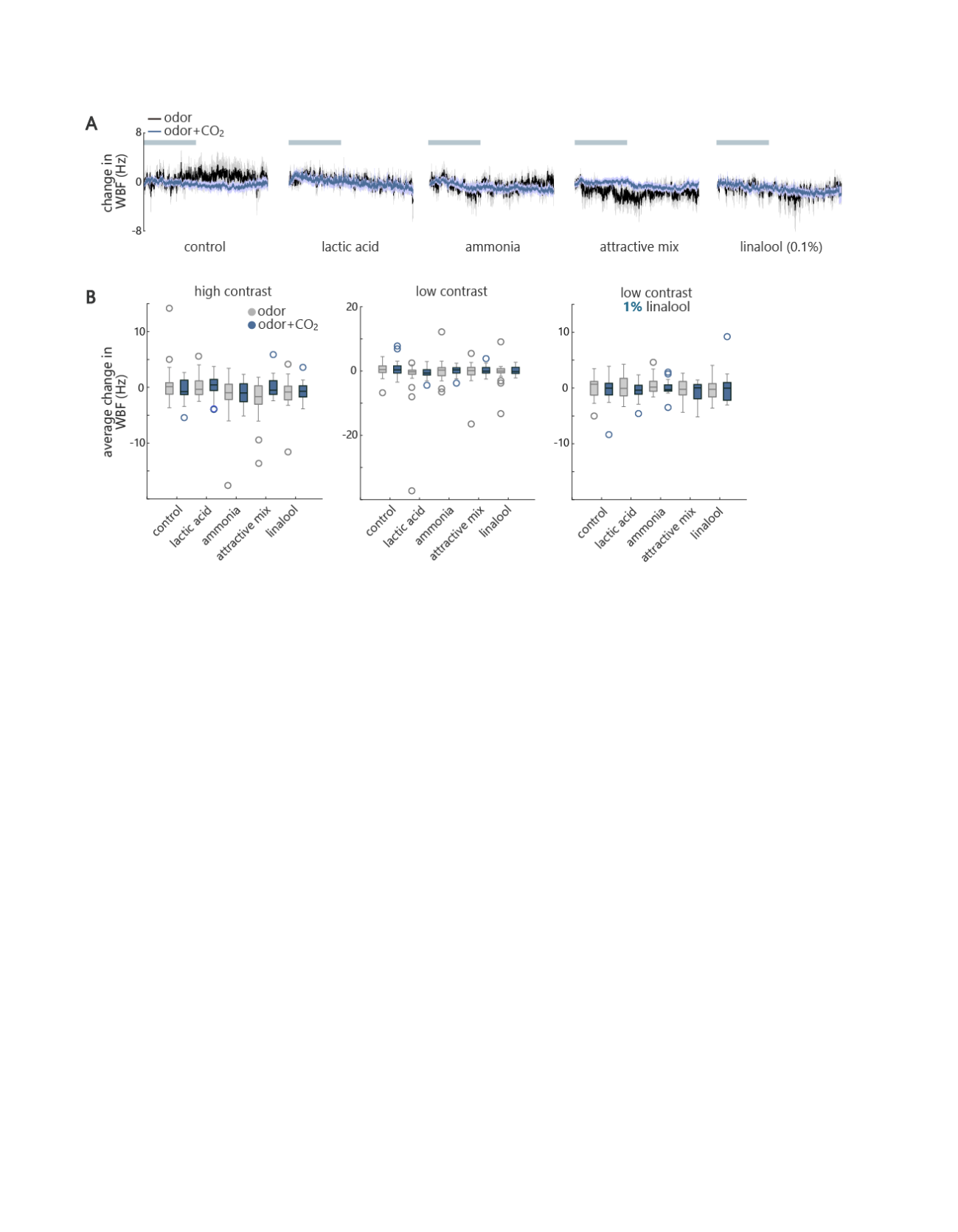


**
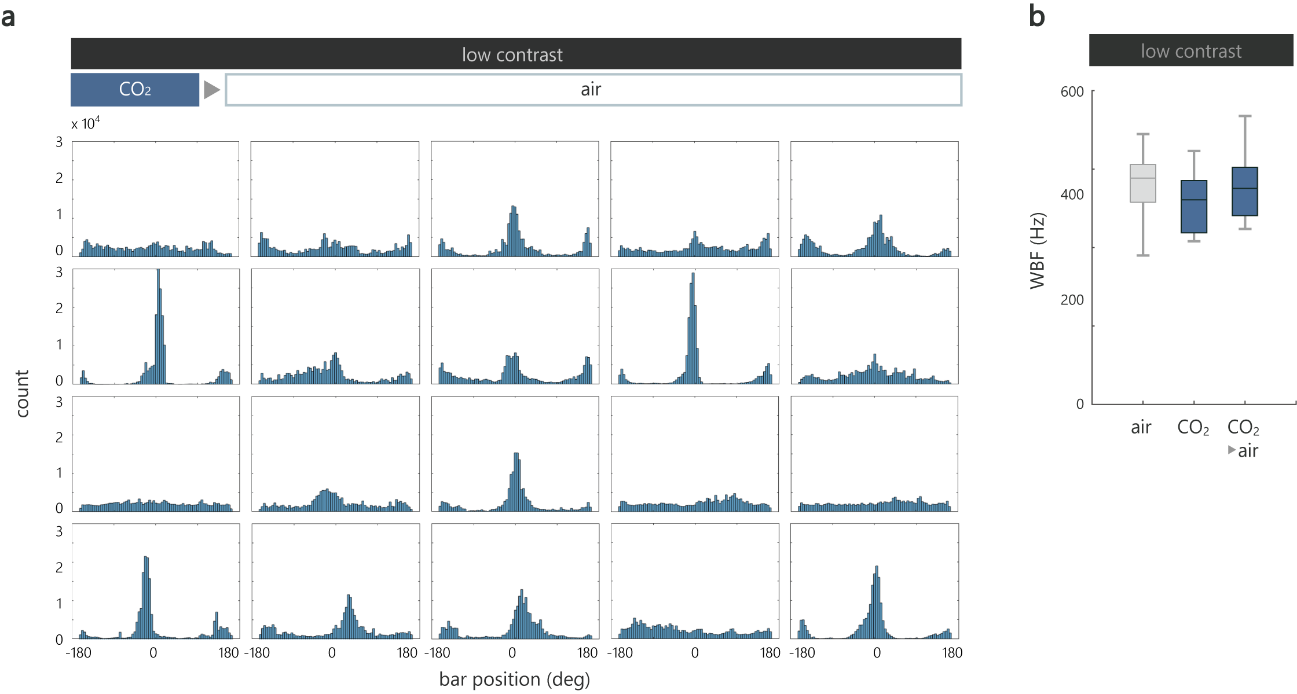
Figure S6. Histogram of bar position under low visual contrast following brief exposure to CO_2_, Related to Fig. 3**

(a) Histogram of bar position for 20 mosquitoes, where CO_2_ was applied for only 10 s just prior to the experiment under low visual contrast. The data are from the same mosquitoes as in Fig. 3g.

(b) WBF did not change under the presence of CO_2_ (p = 0.28, one-way ANOVA, n = 15, 14, and 16 for air, CO_2_, and 10 s CO_2_>air). The data are from the same mosquitoes as in Fig. 3e-3g.


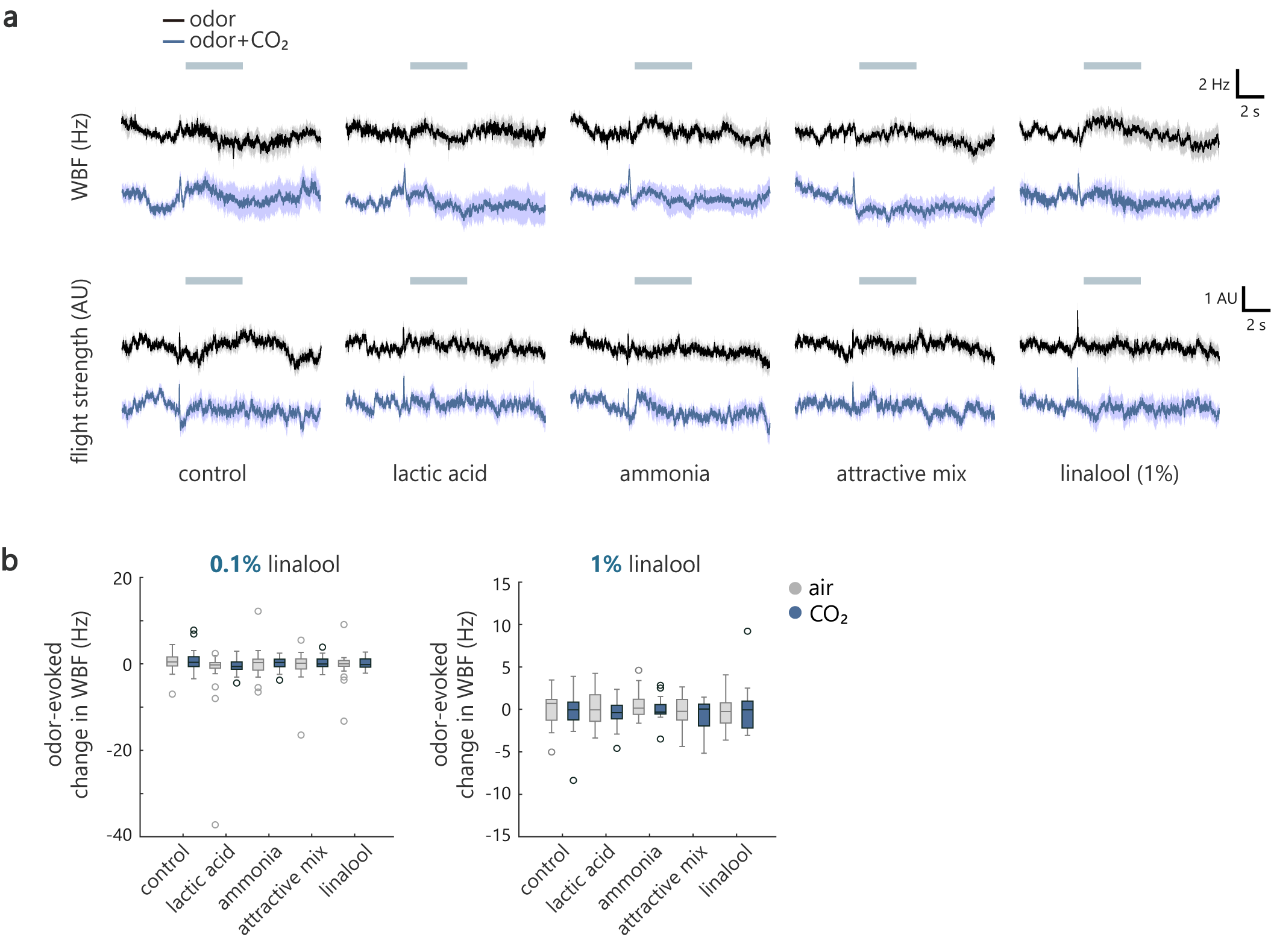


**Figure S7. Odors and CO_2_ do not evoke acute changes in WBF and flight strength, Related to Fig. 4**

(a) Changes in WBF (top two traces) and flight strength (bottom two traces) during odor presentation period with or without the co-application of CO_2_. The data are from the same mosquitoes as in Figs. 4g and 4h. WBF and flight strength do not acutely change in response to odors. Sharp peak preceding the odor onset by ~500 ms likely corresponds to a response to a switching of the solenoid valve that generates a small shock wave. Note that this peak, for example in WBF, is relatively small given that the typical baseline WBF was about 500 Hz.

(b) Average change in WBF during odor application period. The values were similar with or without the co-application of CO_2_ (p = 0.15 and 0.23 for 0.1% and 1% linalool odor sets, repeated measures ANOVA). The data are from the same mosquitoes as in Fig. 4.

Dark and translucent colors represent average and standard error of mean across mosquitoes in (a). Box plots represent median, quartile, nonoutlier range (see Methods for definition), and outliers (circles).

**Supplementary Table 1. Statistical Analyses Conducted in This Study**

This table lists the details of statistical analyses conducted including the type of statistical test, dependent variables, analyzed factors, compared levels for post hoc multiple comparisons, ANOVA conditions (within-subjects repeated measures, between subjects, or interaction), sample size, degrees of freedom, and p values for all the relevant figures.

**Supplementary Table 1. Statistical Analyses Conducted in This Study (Continued)**

**Supplementary Table 1. Statistical Analyses Conducted in This Study (Continued)**

**Supplementary Table 1. Statistical Analyses Conducted in This Study (Continued)**
